# Tools4MSP: an open source software package to support Maritime Spatial Planning

**DOI:** 10.7717/peerj-cs.165

**Published:** 2018-10-01

**Authors:** Stefano Menegon, Alessandro Sarretta, Daniel Depellegrin, Giulio Farella, Chiara Venier, Andrea Barbanti

**Affiliations:** Institute of Marine Sciences, National Research Council, Venice, Italy

**Keywords:** Python, SDI, Tools4MSP software, Open Source, Cumulative Effects Assessment, Maritime spatial planning, GeoNode

## Abstract

This paper presents the Tools4MSP software package, a Python-based Free and Open Source Software (FOSS) for geospatial analysis in support of Maritime Spatial Planning (MSP) and marine environmental management. The suite was initially developed within the ADRIPLAN data portal, that has been recently upgraded into the Tools4MSP Geoplatform (data.tools4msp.eu), an integrated web platform that supports MSP through the application of different tools, e.g., collaborative geospatial modelling of cumulative effects assessment (CEA) and marine use conflict (MUC) analysis. The package can be used as stand-alone library or as collaborative webtool, providing user-friendly interfaces appropriate to decision-makers, regional authorities, academics and MSP stakeholders. An effective MSP-oriented integrated system of web-based software, users and services is proposed. It includes four components: the *Tools4MSP Geoplatform* for interoperable and collaborative sharing of geospatial datasets and for MSP-oriented analysis, the *Tools4MSP package as stand-alone library* for advanced geospatial and statistical analysis, the *desktop applications* to simplify data curation and the *third party data repositories* for multidisciplinary and multilevel geospatial datasets integration. The paper presents an application example of the Tools4MSP GeoNode plugin and an example of Tools4MSP stand-alone library for CEA in the Adriatic Sea. The Tools4MSP and the developed software have been released as FOSS under the GPL 3 license and are currently under further development.

## Introduction

Management and planning of the marine environment require a coordinated development of socio-economic activities, while ensuring a sustainable use of marine resources using an ecosystem-based approach (European Union, 2014; Center for Ocean Solutions, 2011; Douvere, 2008). In the last decade, practical tools to support the implementation of the various steps of Maritime Spatial Planning (MSP) have been developed in various contexts and also analysed to evaluate their usability for different planning purposes (Stelzenmüller et al., 2013; Pınarbaşı et al., 2017).

An extended analysis performed by the “EBM tools network” (https://ebmtoolsdatabase.org/) in support of Ecosystem Based Management has recollected and classified various tools, with respect to types, costs, skills, data and technological requirements. Two categories of tools have emerged in the recent years as analytical support to decision-makers: the sea use conflict analysis and the cumulative impacts assessment.

Sea use conflict analysis has been extensively applied in different geographical contexts (Hadjimitsis et al., 2015; White, Halpern & Kappel, 2012) to investigate and spatially identify conflicts between coastal and marine activities for current conditions and for the comparison of possible future scenarios. In particular, the COEXIST project developed the GRID tool (Georeferenced Interactions Database; Gramolini et al., 2010) to analyse the level of coexistence among uses, depicting areas where different sectors more likely overlap in space and time.

In parallel, various authors proposed methodologies to create cumulative impact maps to reconnect effects of human uses of the sea on environmental components, starting from the methodology firstly introduced by Halpern et al. (2008) at global scale, then implemented in several marine regions, such as Baltic Sea (Korpinen, Meidinger & Laamanen, 2013), North Sea (Andersen et al., 2013), Adriatic Sea (Depellegrin et al., 2017) and at regional scale (Barbanti et al., 2017a; Barbanti et al., 2017b). In particular, Stock (2016) developed an open source software for mapping human impacts on marine ecosystems.

The MSP process involves several user categories, from data producers (e.g., domain experts like ecologists and modellers) to stakeholders and planners. It requires a solid command of geographical information to create a more comprehensive understanding of coastal and marine areas and to support management and planning policies. The development and implementation of Spatial Data Infrastructures (SDI) at multiple levels (i.e., local, regional, national and global) matches the need to make geographical data more accessible and interoperable (Georis-Creuseveau, Claramunt & Gourmelon, 2016). However, various authors (Maguire & Longley, 2005) have highlighted the importance of the integration of Geoportals in the context of SDIs and the role of a user-driven and community-based development as fundamental aspects for an effective and efficient use of the resources (De Longueville, 2010; Georis-Creuseveau, Claramunt & Gourmelon, 2016).

This research presents components and functionalities of the Tools4MSP software package, a Python-based Free and Open Source Software (FOSS) which implements a marine use conflict (MUC) analysis module based on the COEXIST methodology (Gramolini et al., 2010) and a cumulative effects assessment (CEA) module based on the methodology developed in Menegon et al. (2018a). Its implementation in the context of a collaborative Geoplatform supporting MSP and environmental management and its utilization as stand-alone library for cumulative effects assessment (CEA) is tested for the Adriatic Sea.

## Background

### MUC and CEA analysis

The Maritime Use Conflict (MUC) tool implements the COEXIST methodology to estimate the spatial distribution of the conflicts between sea uses. The inputs of the tool are: (i) the area of analysis; (ii) the grid cell resolution; (iii) layers of presence/absence for each human use present in the area (e.g., location of aquacultures, location of oil and gas platforms); (iv) an expert based characterization for each human use through four attributes (vertical scale, spatial domain, temporal domain and mobility). According to the attributes of each use three pre-defined rules, included in the COEXIST methodology are dynamically applied to estimate the potential conflict score for each pair of uses. The potential score varies from 0 (no conflict) to 6 (very high conflict). Afterwards, the area of analysis is subdivided into regular grid cells according to the specified resolution and, on each cell, information about spatial overlapping human uses are extracted. Finally, on each cell the total MUC score is calculated summarizing the potential conflict score for each pair of overlapping uses. The main output is a geospatial distribution of MUC score over the entire area of analysis. For a detailed explanation of the rules and algorithm we refer to Gramolini et al. (2010) and Barbanti et al. (2015).

The Cumulative Effects Assessment (CEA) tool implements the methodology described in Menegon et al. (2018a). Formally, we consider “*CEA as a systematic procedure for identifying and evaluating the significance of effects from multiple pressures and/or activities on single or multiple receptors. CEA provides management options, by quantifying the overall expected effect caused by multiple pressures and by identifying critical pressures or pressure combinations and vulnerable receptors. The analysis of the causes (source of pressures), pathways, interactions and consequences of these effects on receptors is an essential and integral part of the process*” (Judd, Backhaus & Goodsir, 2015).

The inputs of the Tools4MSP CEA tool are: (i) the area of analysis; (ii) the grid cell resolution; (iii) layers representing intensity or presence/absence of human uses (e.g., intensity of fishery and maritime transport, presence of aquacultures and oil & gas platforms); (iv) layers representing intensity or presence/absence of environmental components (e.g., seabed habitats, probability of presence of nursery habitats, probability of presence of marine mammals); (v) use-specific relative pressure weights and distances of pressure propagation; (vi) environmental component sensitivities related to specific pressures or more general ecological models that describe the response of the environmental components to a specific pressure. Similarly to the MUC tool, the area of analysis is subdivided into regular grid cells. Then, on each cell, information about the presence of human uses and environmental components are extracted. Afterwards, the geospatial layers on human uses are propagated and combined to estimate the geospatial distribution of different pressures (e.g., marine litter, underwater noise, abrasion) for the entire analysis area. Finally, the geospatial distribution of single and cumulative effects and impacts are estimated combining together the pressure layers and the environmental components layers through a sensitivity score.

### The MSP-oriented integrated system

**Figure 1:**
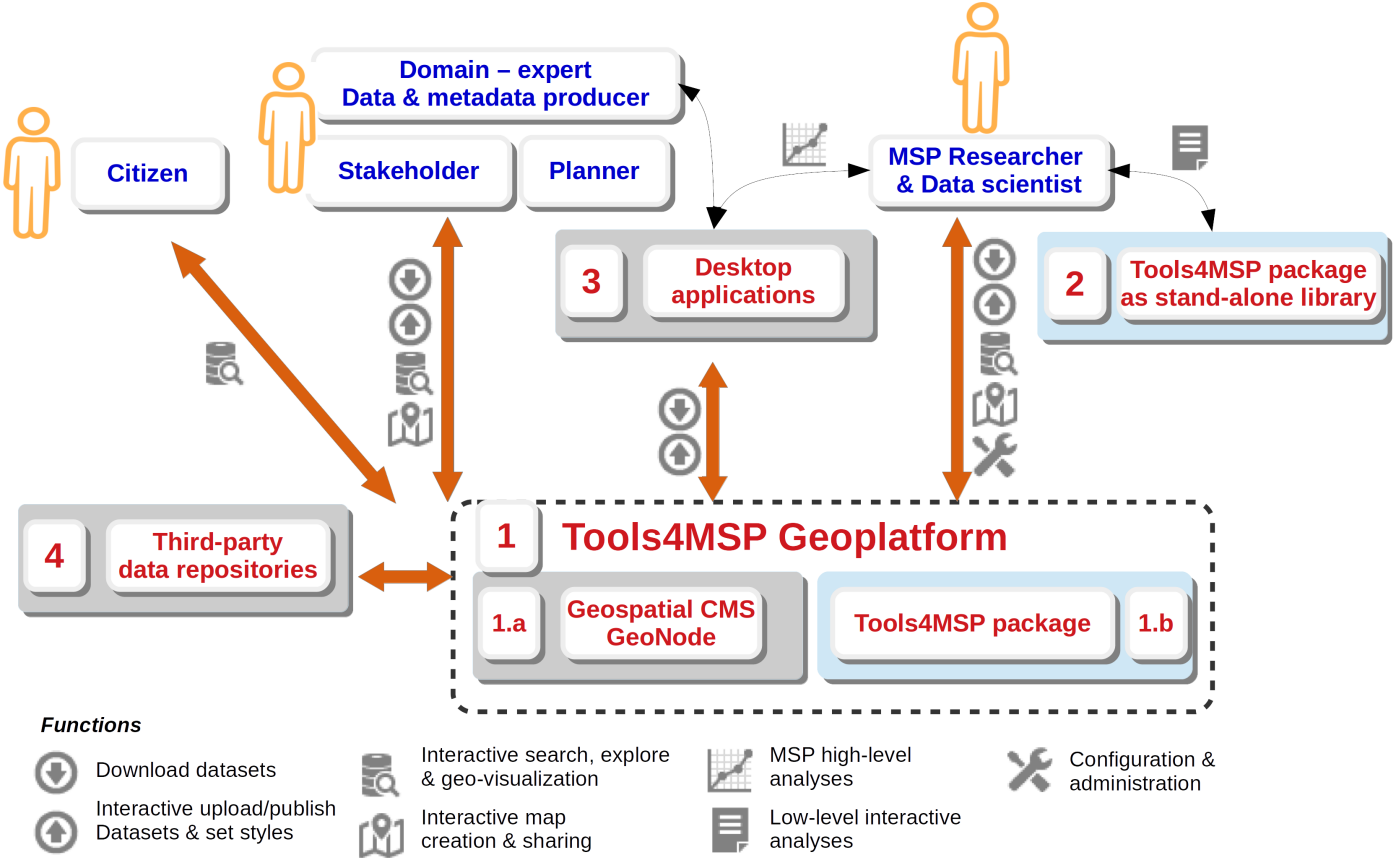
Interaction diagram between the Tools4MSP Geoplatform, external applications and potential end-users. Component 1.b and 2 (blue background) are developed and released with the Tool4MSP package.

In Fig. 1 the interactions between the Tools4MSP Geoplatform, external application and potential end-users are presented. As a whole, they represent an integrated system of software, users and web services capable to effectively support MSP activities. Overall, the system can be described by four components:

#### Component 1: Tools4MSP Geoplatform

The geoplatform is a community-based integrated web application. Data are managed in a SDI over the entire workflow, from the collaborative upload in a web portal, to the creation of metadata, the choice of appropriate visual encodings, the composition of maps, the set up of use cases and the elaboration through specific modules producing final maps and descriptive reports. Internally, the Tools4MSP Geoplatform is divided into two sub-components: the Geospatial Content Management System (CMS; 1.a) and the Tools4MSP package (1.b). A more detailed description of the Geoplatform is given in the next section.

#### Component 2: Tools4MSP package as stand-alone library

The package can also be downloaded and used as stand-alone library independently from the GeoNode software. The library can be efficiently used through Jupyter Notebook (Kluyver et al., 2016; https://jupyter.org/), a web-based computational environment, which provides one of the most convenient user interfaces for interactive analysis (McGibbon et al., 2015; Shen, 2014). The software allows the authoring of shareable and reproducible notebook documents which allow a combination of input code, rich media representations of the output results, explanatory text, mathematics, images forming a rich computational narrative (Kluyver et al., 2016). Regarding the Tools4MSP development, the Jupyter Notebook supports rapid prototyping of new features, libraries testing and advanced analysis of case studies (Fig. 1, components 2).

#### Component 3: Desktop applications

Spatial layers and maps managed in the Geoplatform can be downloaded in different formats directly from the web interface or can be reused in desktop GIS applications for further investigation by connecting to standard web services. Data curation can be also improved using the desktop GIS QGIS (QGIS Development Team, 2018) together with the GeoServer explorer plugin (Boundless, 2018). This plugin eases the publication, upload and visual encoding of data and layers, allowing collaborators and domain experts to better contribute to the update and maintenance of the Geoplatform content.

#### Component 4: Third party data repositories

MSP-related workflows need relevant and updated data to be analysed and processed, the interaction between this component and the Tools4MSP Geoplatform highlights the ability of the system to integrate data from other data portals and SDIs, such as SHAPE Adriatic Atlas (http://atlas.shape-ipaproject.eu/), EMODnet portals (http://www.emodnet.eu/), EEA services (https://www.eea.europa.eu/data-and-maps), CoCoNet web GIS (http://coconetgis.ismar.cnr.it/) or the European Atlas of the Seas (https://ec.europa.eu/maritimeaffairs/atlas/maritime_atlas). All these portals use interoperable OGC-compliant web services to exchange spatial information; based on GeoNode features, the Tools4MSP Geoplatform allows users to display external layers (e.g., served from remote Web Map Services; OGC, 2006) and enriches its catalogue with relevant data through the harvesting of standard web services (GeoNode remote services). The creation and maintenance of this network of collaborations allow to harmonize existing multiple efforts and improve the availability of spatial datasets for users interested in MSP-related information.

### The Tools4MSP Geoplatform

**Figure 2:**
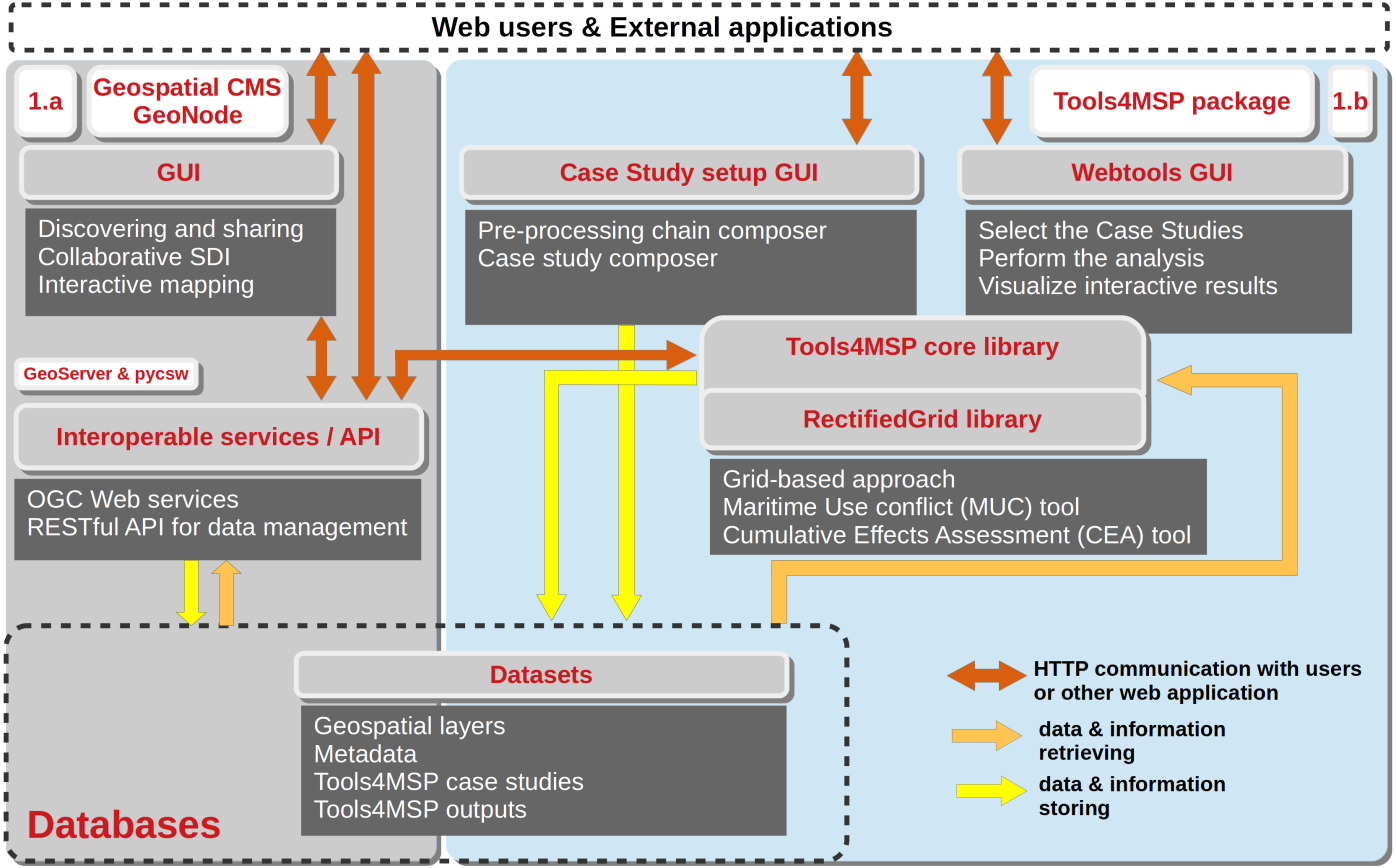
Implementation architecture of the Tools4MSP collaborative Geoplatform.

In Fig. 2 the detailed implementation architecture of the Tools4MSP Geoplatform is presented. The Geospatial CMS is the core of the system and is based on the GeoNode (GeoNode, Developers and Contributors Team, 2018) software, a Django-based web platform for developing community-based SDI. GeoNode facilitates the upload and management of geospatial datasets, making them discoverable and available via standard Open Geospatial Consortium (OGC) protocols and web mapping applications. GeoNode also allows users to automatically upload, describe and share the outputs produced by the Tools4MSP package.

The Tools4MSP package is Python-based open source software available on github (Menegon, 2018a). The Tools4MSP core library implements the algorithms for CEA and MUC geospatial modelling and the base functionalities to read the input geospatial datasets and configurations, to apply data transformation operations (such as normalization, aggregation, reclassification, gaussian convolution, geospatial filtering) and to manage and write model output results. Tools4MSP adopts a grid-based approach for efficient numerical computation of the geospatial models. The grid-based functionalities are provided through the general purpose *RectifiedGrid* library (Menegon, 2018b), that ensures direct integration of a multitude of different datasets and facilitates data preparation procedures. It simplifies the access and rasterization of multi-format geospatial data (environmental and anthropogenic datasets) and performs arithmetic and transformation operations on raster map layers. RectifiedGrid combines several FOSS Python projects: (1) NumPy and SciPy for efficient numerical computation (Van der Walt, Colbert & Varoquaux, 2011) and (2) rasterio and fiona (Mapbox, Rasterio Development Team, 2018; Fiona, Developers and Contributors team, 2018) for multi-format raster and vector data access through the Geospatial Data Abstraction Library (GDAL; Warmerdam, 2008). Interactive graphics for visualization of output results are based on the Bokeh visualization library (Bokeh Development Team, 2018).

Different users can have different modes of interaction with the Geoplatform: administrators perform the case study setup, by specifying the connectors to the geospatial repositories and defining the pre-processing chain for environmental and human use layers harmonization and utilization in the case study (Fig. 2, Case Study setup GUI).

A broad user community composed by decision-makers, planners, academics, research institutions, MSP stakeholders and the general public can use the case study configuration to run the build-in version of the Tools4MSP library implemented in the Geoplatform (Fig. 2, Webtool GUI). More in detail, the Tools4MSP Webtool GUI implements a four step workflow: (Step 0) Webtool selection (MUC or CEA); (Step 1) case study selection, choosing from a pre-set of case studies; (Step 2) case study configuration, optionally outlining a custom subregion of analysis or a custom combination of human uses, pressures, and environmental components; (Step 3) generation of geospatial and statistical outputs. The outputs are published in GeoNode and accessible through standard interoperable services. The produced reports, graphics and statistical outputs are archived in a dedicated data store catalogue and can be further visualized and re-used also by non technical stakeholders. In the results section an application of the Tools4MSP GeoNode plugin for case study setup and CEA analysis in the Adriatic Sea is provided.

## Results

At the current stage, the Tools4MSP modelling framework has been applied in various areas of interest in the Adriatic Sea, such as entire Adriatic Sea (Depellegrin et al., 2017), Italian Adriatic Sea (Menegon et al., 2018a) and regional scale analysis for the Northern Adriatic Sea (Menegon et al., 2018b) and Emilia-Romagna Region (Barbanti et al., 2017a; Barbanti et al., 2017b). In the following section results for Tools4MSP GeoNode plugin and stand-alone library for the Adriatic Sea will be presented.

### Application of Tools4MSP GeoNode plugin

The Tools4MSP GeoNode plugin implements two sets of interfaces: the case study setup GUI and the Webtool GUI.

**Figure 3:**
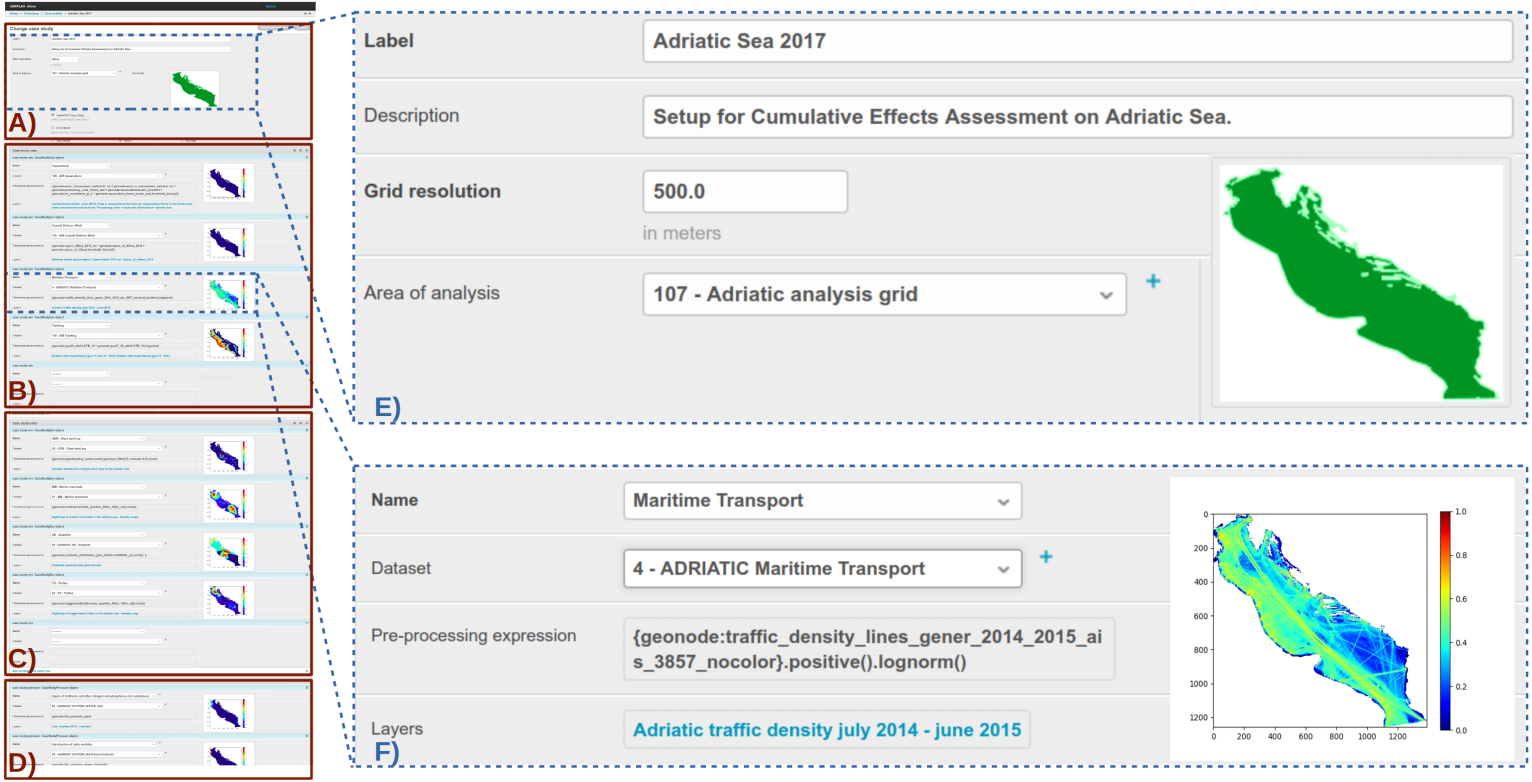
Example of Case Study setup Graphical User Interface.

In Fig. 3 an example of GUI illustrating a case study setup is presented. The interface is intended for administrators and was designed to facilitate the configuration of case study input parameters. The GUI is organized in 4 sections: (i) basic parameters (Fig. 3A); (ii) human uses (Fig. 3B); (iii) environmental components (Fig. 3C); (iv) pressures (Fig. 3D). In the example the case study named “Adriatic Sea 2017” has a grid resolution of 500 m and the area of analysis is the Adriatic Sea (Fig. 3E). Figure 3F shows the expanded view of geospatial dataset set up for “Maritime Transport”. Specific data transformation operations have been configured through the “Pre-processing expression” field. The expression is written with a Python-based syntax that allows the user to select and combine one or more layers, apply filters, apply masking conditions, perform grid-cell-based arithmetic and other data transformations (e.g., normalization, logarithmic scaling, gaussian convolution). The resulting geospatial dataset is shown as thumbnail directly into the case study setup GUI (Fig. 3F).

The example reported in Fig. 3F refers to a human use (Maritime Transport) but is equally valid to environmental components or pressures.

The Webtool GUI is the standard interface allowing non-technical users (e.g., decision-makers, MSP stakeholders, planners) to perform CEA and MUC analyses starting from the pre-set case studies.

**Figure 4:**
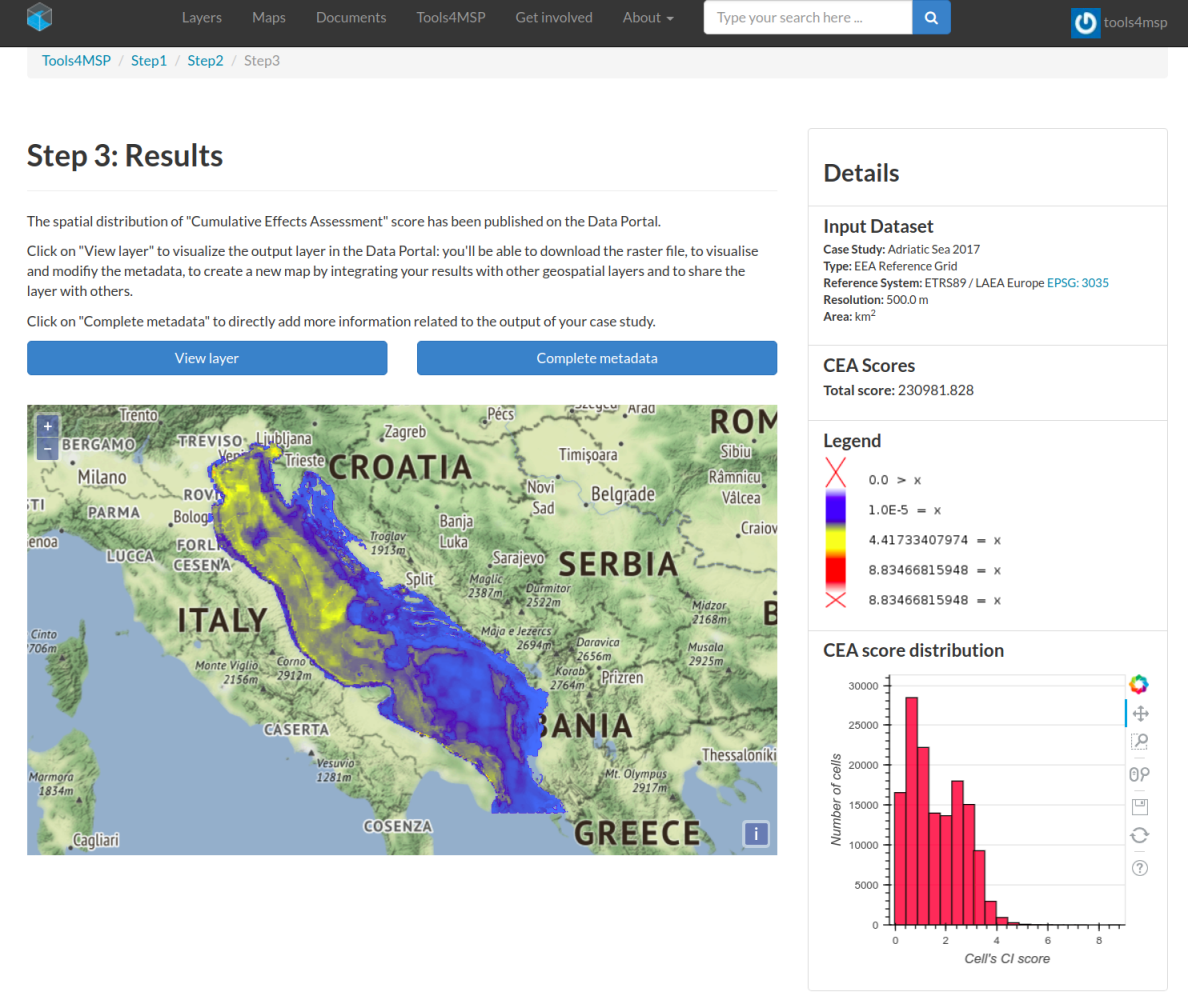
Example of Graphical User Interface presenting geospatial and statistical results.

The Webtool GUI implements a four-steps workflow: (Step 0) Webtool selection; (Step 1) Case study area selection; (Step 2) Study area selection & Dataset configuration; (Step 3) Geospatial and statistical outputs. An example of the outputs (Step 3: Results) for the case study “Adriatic Sea 2017” is shown in Fig. 4. Through the GUI, the geospatial distribution of CEA for the analysis area as well as the statistical outputs can be explored and downloaded.

A complete example of MUC and CEA analysis through the Tools4MSP webtool GUI including a more in-depth investigation on its strengths and limitations in support of Maritime Spatial Planning is available in Menegon et al. (2018b).

### Application of Tools4MSP package as stand-alone library

In this section we present a cumulative effects assessment based on the stand-alone Tools4MSP package applied for the Adriatic Sea. The case study set up has been downloaded from the Tools4MSP Geoplatform. It consists of a directory named “adriatic_sea” containing the input geospatial layers related to human uses, environmental components and pressures and environmental component sensitivities. An example of the case study set up is released within the Tools4MSP software package (https://github.com/CNR-ISMAR/tools4msp/tree/master/data/demo_case_study) and is available for test and demo purposes.

**Figure 5:**
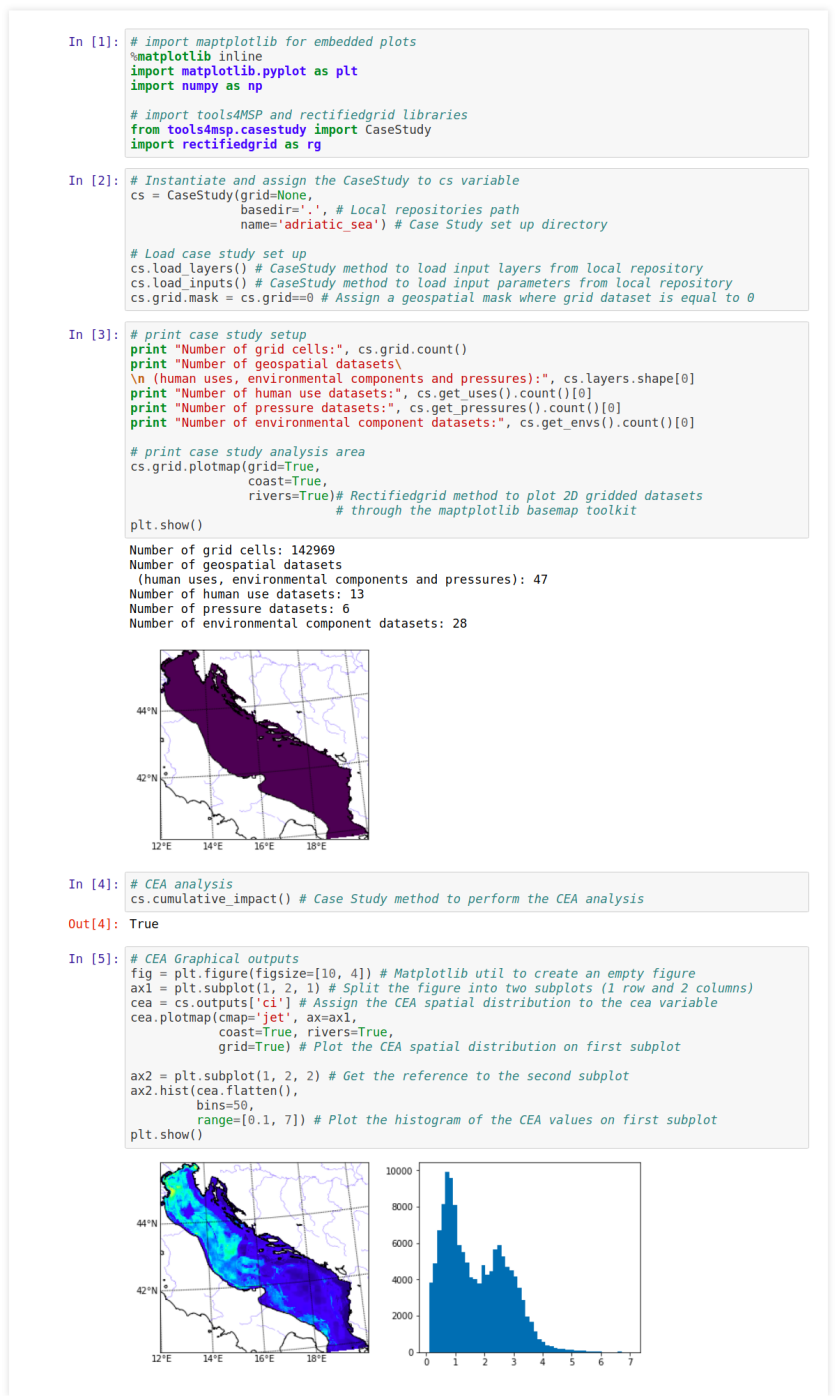
Workflow for Tools4MSP stand-alone library application using Jupyter computational environment.

The case study is presented using a workflow implemented through the Jupyter computational environment including the following steps (Fig. 5):

• *In [1]* Import libraries: Tools4MSP *CaseStudy* class, *rectifiedgrid* using the *rg* alias, *matplotlib* and *numpy. CaseStudy* is a comprehensive class that provides the methods to setup a case study (e.g., specify datasets and input parameters) or alternatively to load a predefined case study, perform the analysis and manage the results.
• *In [2]* Load predefined case study for Adriatic Sea (1 km ×1 km resolution). After instantiating the *CaseStudy* class specifying the “adriatic_sea” case study, the methods *load_layers* and *load_inputs* are called in order to import the environmental, pressure and human use layers (including the layer metadata) and the input parameters respectively (e.g., sensitivity scores).
• *In [3]* Print example case study setup providing general information on spatial extent (in number of cells) and number of available layers (geospatial datasets). The input geospatial layers and the area of analysis layer (*.grid* parameter) are instances of the *RectifiedGrid* class. *RectifiedGrid* is the main class provided by the *rectifiedgrid* library and it is designed to represent and manipulate 2D georeferenced data arrays. *RectifiedGrid* provides the “.*plotmap*” method to plot the layer and other information (e.g., coastline, rivers) on a map.
• *In [4]* CEA analysis function. The *CaseStudy* class exposes the “.cumulative_impact” method to perform a Cumulative Effects Assessment (Menegon et al., 2018a).
• *In [5]* CEA geospatial results and graphical outputs for the Adriatic Sea. The map of the CEA result is visualized using the “.plotmap” method in combination with the distribution of CEA values of the grid cells.

### Software details

In Table 1 the summary of the main characteristics and requirements of *RectifiedGrid* and Tools4MSP is presented.

**Table 1:** Software characteristics, requirements and availability for RectifiedGrid library and Tools4MSP package.

	**RectifiedGrid**	**Tools4MSP**
Language	Python	Python
Operating system	Platform-independent; requires Python distribution	Platform-independent; requires Python distribution
Dependencies	Numpy, GeoPandas, Scipy, Rasterio, Fiona, Shapely, Rtree, Affine, Matplotlib, GDAL	RectifiedGrid, Bokeh, GeoNode (for plugin usage)
Software location	DOI: 10.5281/zenodo.1185428	DOI: 10.5281/zenodo.1186160
Code repository	https://github.com/CNR-ISMAR/rectifiedgrid	https://github.com/CNR-ISMAR/tools4msp
License	GPL 3	GPL 3

## Discussion and Conclusions

This paper presents architecture, implementation and practical application of an MSP-oriented software package named Tools4MSP. The tool is presented as GeoNode plugin and as a stand-alone library determining different levels of usability by different user groups.

As a plugin, Tools4MSP supports a wide user community that facilitates the implementation of collaborative analyses improving the reusability and sharing of the result outputs. The integration within a Geospatial CMS allows to manage the entire processing data workflow, from the collaborative upload in a web portal, to the creation of metadata, the choice of appropriate visual encodings, the composition of maps, the set up of use cases and the elaboration through specific modules producing final maps and descriptive reports. The usage of the plugin is particularly suitable as it provides a user-friendly interface appropriate to decision-makers, regional authorities, academics and MSP stakeholders (e.g., fishers, eNGOs, industry).

The plugin eases data transformation operations reducing the need of manual data preparation and standardization procedures. Furthermore, archiving the pre-processing expressions in combination with the case study makes the transformation of the input data more explicit and the entire process more transparent and replicable.

As stand-alone library, Tools4MSP requires advanced programming skills for its usage, but provides more flexible integration with other libraries and Python packages also outside Tools4MSP modelling framework. It is particularly suitable for planning authorities seeking advanced modelling procedure for MSP/ICZM and management purposes. The scientific community, consultancies and programmers can use and further develop the library for different research objectives and integration into Decision-Support-Systems.

Compared to other existing decision support tools in MSP, the Tools4MSP “*approach*” is more flexible as it (1) incorporates a multi-objective toolset (CEA and MUC) which can be extended for other analysis purposes (e.g., scenario analysis, comparative MSP or ecosystem services assessment); (2) it enables management and treatment of different datasets and formats and (3) it provides different levels of usability ranging from experienced modellers to more user-friendly modelling through GUIs.

The tools CEA and MUC models implemented can support the development of maritime spatial plans within the implementation process of the MSP Directive (2014/89/CE) in various case study areas and marine waters in the Mediterranean Sea and beyond. The package is regularly upgraded within the Tools4MSP Geoplatform (data.tools4MSP.eu) including ongoing implementations of pressure specific analysis of CEA, CEA backsourcing (CEA-B; Menegon et al., 2018a) and the integration of marine ecosystem services oriented analysis of anthropogenic threats (MES-Threat) in support of environmental management and resource restoration (Depellegrin & Blažauskas, 2013; Menegon et al., 2018b). The Tools4MSP software package was used in ADRIPLAN (ADRiatic and Ionian maritime spatial PLAnning) and RITMARE (Ricerca ITaliana per il MARe) projects and is currently implemented by various research communities within ongoing European Projects around the Mediterranean, such as SUPREME (Supporting maritime spatial Planning in the Eastern Mediterranean) or PORTODIMARE (geoPortal of Tools & Data for sustainable Management of coAstal and maRine Environment).

## Additional Information and Declarations

### Data Availability

The following information was supplied regarding data availability: Stefano Menegon. (2018, February 28). Tools4MSP: Geospatial tools to support Maritime Spatial Planning (Version 1.0.0-beta.3). Zenodo. DOI 10.5281/zenodo.1186160. Stefano Menegon. (2018, February 27). RectifiedGrid: Geospatial python library for grid-based analyses (Version 1.0.0-beta.4). Zenodo. DOI 10.5281/zenodo.1185428.
